# A Decision-Making Method for Photon/Proton Selection for Nasopharyngeal Cancer Based on Dose Prediction and NTCP

**DOI:** 10.3390/cancers17162620

**Published:** 2025-08-11

**Authors:** Guiyuan Li, Xinyuan Chen, Jialin Ding, Linyi Shen, Mengyang Li, Junlin Yi, Jianrong Dai

**Affiliations:** National Cancer Center/National Clinical Research Center for Cancer/Cancer Hospital, Chinese Academy of Medical Sciences and Peking Union Medical College, Beijing 100021, China

**Keywords:** decision-making, deep learning, NTCP, nasopharyngeal cancer, proton therapy, photon therapy

## Abstract

**Introduction**: Decision-making regarding radiotherapy techniques for patients with nasopharyngeal cancer requires a comparison of photon and proton plans generated using planning software, which requires time and expertise. We developed a fully automated decision tool to select patients for proton therapy that predicts proton therapy (XT) and photon therapy (PT) dose distributions using only patient CT image data, predicts xerostomia and dysphagia probability using predicted critical organ mean doses, and makes decisions based on the Netherlands’ National Indication Protocol Proton therapy (NIPP) to select patients likely to benefit from proton therapy. **Methods**: This study used 48 nasopharyngeal patients treated at the Cancer Hospital of the Chinese Academy of Medical Sciences. We manually generated a photon plan and a proton plan for each patient. Based on this dose distribution, photon and proton dose prediction models were trained using deep learning (DL) models. We used the NIPP model to measure xerostomia levels 2 and 3, dysphagia levels 2 and 3, and decisions were made according to the thresholds given by this protocol. **Results**: The predicted doses for both photon and proton groups were comparable to those for manual plan (MP). The Mean Absolute Error (MAE) for each organ at risk in the photon and proton plans did not exceed 5% and showed a good performance of the dose prediction model. For proton, the normal tissue complication probability (NTCP) of xerostomia and dysphagia performed well, *p* > 0.05. There was no statistically significant difference. For photon, the NTCP of dysphagia performed well, *p* > 0.05. For xerostomia *p* < 0.05 but the absolute deviation was 0.85% and 0.75%, which would not have a great impact on the prediction result. Among the 48 patients’ decisions, 3 were wrong, and the correct rate was 93.8%. The area under curve (AUC) of operating characteristic curve (ROC) was 0.86, showing the good performance of the decision-making tool in this study. **Conclusions**: The decision tool based on DL and NTCP models can accurately select nasopharyngeal cancer patients who will benefit from proton therapy. The time spent generating comparison plans is reduced and the diagnostic efficiency of doctors is improved, and the tool can be shared with centers that do not have proton expertise. **Trial registration**: This study was a retrospective study, so it was exempt from registration.

## Simple Summary

Decision-making about radiotherapy techniques for nasopharyngeal cancer using plans generated by planning software is time-consuming and requires specialized knowledge. The aim of our retrospective study was to develop a tool that can quickly and accurately provide decision-making results. We used dose prediction and normal tissue complication probability model to make photon-proton selection for 48 patients, achieving an accuracy rate of 93.8%. This tool can identify patients who will benefit from proton therapy, reducing decision-making time and improving doctors’ efficiency. This advanced tools can also be transferred to lower-level radiotherapy institutions to improve their diagnosis and treatment level.

## 1. Introduction

Nasopharyngeal carcinoma (NPC) is a rare malignancy in most parts of the world, but it is rather prevalent in southern China [1]. Radiation therapy (RT) is a common approach for NPC patients [2]. Head and neck radiotherapy requires irradiation of target volumes to high dose levels in the proximity of many normal tissues, especially NPC [3]. Advances in radiation therapy, such as intensity-modulated radiation therapy (IMRT) and the Volumetric Modulated Arc Therapy (VMAT), have allowed high-dose delivery to tumors while sparing normal tissues and therefore reducing acute and late radiation-induced toxicities [4,5,6]. However, toxicity rates are still related to RT, especially for advanced clinical stages, with substantial effects on quality of life [7,8,9,10].

Proton radiotherapy has important advantages compared to the currently used photons due to its unique dose deposition characteristics [11]. In particular, compared with advanced photon RT techniques such as VMAT, intensity-modulated proton therapy (IMPT) can reduce radiation-induced side-effects to organs at risk (OARs) and maintain highly conformal target coverage [12].

However, proton therapy (PT) equipment is complex and carries high maintenance costs, resulting in relatively high treatment costs, which makes it difficult for many patients, especially those with limited financial means, to afford PT. At present, PT systems are only available in some large hospitals or specialized treatment centers, which limits the access of patients to PT, especially in areas with relatively poor medical resources. Although PT is superior to conventional radiotherapy in reducing normal tissue damage, there is still a certain risk of side effects. PT should therefore be reserved for patients who are likely to benefit the most [13].

Recently, the mainstream approach to selecting the patients who could benefit most from PT is by using a model-based prediction methodology. Langendijk et al. [11] established a model-based decision-making method for head and neck cancer in 2013. The model-based approach was approved by the Health Council in the Netherlands and eventually formed the Dutch standard guide National Indication Protocol Proton therapy (NIPP) [14]. The methodology could be based on the dose reduction to relevant OARs resulting from plan comparison between photon therapy (XT) and PT, translated into a clinically relevant benefit, estimated in terms of reduced risk of side effects by means of normal tissue complication probability (NTCP) models [15]. Many studies have used the NTCP model to evaluate complications after radiation therapy [16,17,18,19]. The difference between these risks (NTCP_XT_ − NTCP_PT_) is the ΔNTCP, i.e., the expected reduction in complications because of a reduction in dose to healthy tissues with PT as compared to XT. If the ΔNTCP reaches a certain threshold for a certain complication, then PT is considered [20].

This study proposed a novel method that combined dose prediction and NTCP model to develop an efficient and rapid decision-making strategy for photon and proton therapy of NPC. First of all, we used advanced deep learning method for dose prediction, which can automatically and accurately obtain the expected dose distribution without manual planning. Then, the NTCP values for xerostomia and dysphagia were automatically calculated based on photon and proton dose predictions. Finally, NIPP model was used to make decisions and select the treatment technology (proton or photon) suitable for the patient.

## 2. Methods

### 2.1. Patient Selection and Plan

A total of 48 consecutive NPC patients treated between January 2021 and December 2022 have been retrospectively included in this study. The use of patient data for the study was approved by the Institutional Ethical Review Board of the National Cancer Center (NCC). For each patient, a PT plan and XT plan were manually generated by an experienced dosimetrist with the treatment planning system (TPS) Pinnacle16.2. The manual plans (MP) of PT and XT are regarded as reference plans. The PT and XT plans employed the same prescriptions and optimization goals shown in Table 1. All selected patients have three dose prescription levels: 73.92 Gy (PGTV74), 69.96 Gy (PGTV70), and 60.06 Gy (PTV60). The XT plans are planned for volumetric modulated arc therapy (VMAT)with two full coplanar arcs. Proton therapy plans were generated using pencil beam scanning (PBS) technique with four coplanar beams. The gantry angles were 215°, 300°, 60°, and 145°, respectively. Each patient has a CT image and a set of contours for the target volumes (PGTV or PTV) and the OARs: brain stem, spinal cord, parotid, oral cavity, submandibular glands, pharyngeal constrictor muscles inferior (PCM I), middle (PCM M), and upper (PCM U).

### 2.2. Workflow of Decision-Making Method

**Figure 1:**
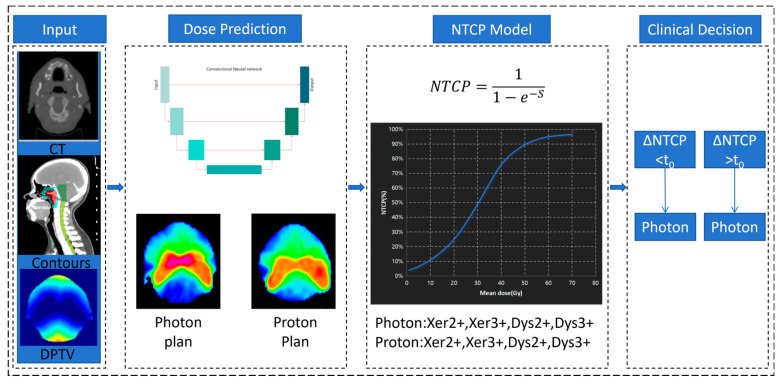
The workflow of the decision-making method. The inputs are the CT image and the contours and distance map (DPTV). Then the photon and proton dose distributions are predicted according to the trained dose prediction deep learning model. The predicted mean dose of OARs is used as an input into the NTCP model to calculate the probability of xerostomia and dysphagia. Finally, decisions were made according to the NIPP decision tree.

Our decision tool mainly consists of dose prediction and NTCP model shown in Figure 1. First, a dose-prediction model was established using the VMAT and IMPT dataset comprising the CT images, contour masks, distance map, and corresponding dose distribution. Second, we input predicted mean doses of OARs into the NTCP model to calculate the probability of xerostomia and dysphagia for XT and PT. Finally, the decision of treatment technology for patients is made according to the decision tree of the Dutch national model NIPP.

### 2.3. Deep Learning-Based Dose Prediction Method

**Figure 2:**
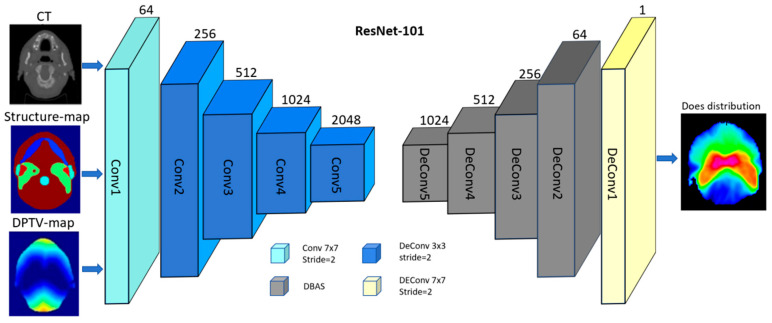
Training workflow based on 101 with three input channels (CT, structure maps, and DPTV maps) of the COM model.

We trained a deep-learning combined model (COM) inspired by the popular NCC architecture [21] to predict the optimal three-dimensional dose distribution, shown in Figure 2. The COM model was to use combined anatomical maps as three channel inputs that included DPTV maps, structure maps, and CT. The DPTV is defined as the minimum distance to PTV surface for each voxel of normal tissue that was outside the PTV but within body range. The outputs were the corresponding dose distribution maps. The main generator of the network is based on Layer 101 of Resnet, which has been introduced in our previous research [22]. Conv1 is a 7 × 7 convolutional layer with 64 filters. A max-pooling operation is then performed for downsamplisng. Conv2, Conv3, Conv4, and Conv5 consist of 3, 4, 23, and 5 deeper bottleneck architectures (DBAs), respectively [23]. Each DBA has three convolutional layers of 1 × 1, 3 × 3, and 1 × 1 and a connection. The output of Conv5 is 1/8 of the original image. Upsampling based on the fractionally-strided deconvolution is used to restore the image resolution. PyTorch 2.3.0 was used to implement the process of model training and testing on a workstation equipped with an Intel^®^ Core i7 CPU (3.4 GHz) and a TITAN XP graphics card. A total of 100 epochs were set for training to ensure loss convergence. An eight-fold cross-validation scheme was implemented. The 48 cases were evenly divided into 8 subsets of 6 patients each; 7 subsets were used for training and the remaining subset for testing.

### 2.4. NTCP Modeling

According to the updated Dutch NIPP, which includes four NTCP models, we calculated the NTCP value for Grade ≥ 2 xerostomia and dysphagia and Grade ≥ 3 xerostomia and dysphagia. In addition to baseline xerostomia and dysphagia complaints, these NTCP models include the Dmean of eight OARs as predictors, including the oral cavity, submandibular, and parotid glands and PCM U, PCM M, and PCM I [14].

The logistics model based on DVH was adopted to calculate NTCP value shown in Equation (1):

(1) $$\mathrm{NTCP}\;=\;\frac{1}{1-e^{-S}}$$

where *S* is calculated according to the Coefficients of the original NTCP models from the NIPP. The S of Xer2+ was calculated as shown in Equation (2):

(2) $$S=-2.2951+0.0996\times (\sqrt{Dmean\;PL}+\sqrt{Dmean\;PR})+0.0182\times Dmean\;Sub$$

where PL is parotid left, PR is parotid right, and Sub is submandibular.

The S of Xer3+ was calculated as shown in Equation (3):

(3) $$S=-3.7286+0.0855\times (\sqrt{Dmean\;PL}+\sqrt{Dmean\;PR})+0.0156\times Dmean\;Sub$$

The S of Dys2+ was calculated as shown in Equation (4):

(4) $$\begin{array}{l} S=-4.0536+0.030\times Dmean\;OC+0.0236\times Dmean\;PCM\;U+0.0095\times Dmean\;PCM\;M+\\ 0.0133\times Dmean\;PCM\;I-0.6281 \end{array}$$

where OC is oral cavity.

The S of Dys3+ was calculated as shown in Equation (5):

(5) $$\begin{array}{l} S=-7.674+0.0259\times Dmean\;OC+0.0203\times Dmean\;PCM\;U+0.0303\times Dmean\;PCM\;M+\\ 0.0341\times Dmean\;PCM\;I+0.0387 \end{array}$$

### 2.5. Decision-Making

**Figure 3:**
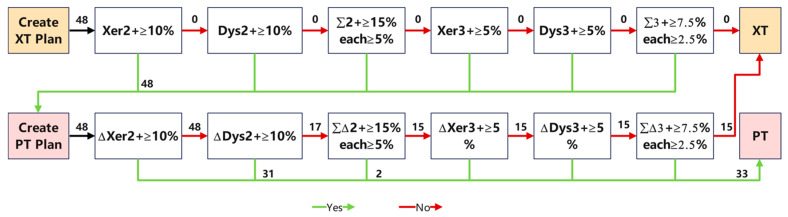
The flowchart for patient selection. The figure marks the screening conditions and the number of people selected for each decision node [13]. Δ = NTCP_XT_ − NTCP_PT_; Σ2+ = (Xer2+) + (Dys2+); Σ3+ = (Xer3+) + (Dys3+); ΣΔ2+ = (ΔXer2+) + (ΔDys2+); ΣΔ3+ = (ΔXer3+) + (ΔDys3+); XT—radiotherapy; PT—proton therapy.

Patients receive an indication for PT if the predicted complication risk for their PT plan is sufficiently lower than the predicted complication risk given their XT plan and if their ΔNTCP (NTCP_XT_ − NTCP_PT_) is sufficiently large, considering the complications and thresholds stated in the NIPP. NTCP for grade 2 and 3 xerostomia and dysphagia at 6 months after the treatment were calculated using the models adopted by the Dutch Radiation Oncology Society. Figure 3 is the decision flow chart given in this study based on the NIPP decision tree. If ΔXer2+ ≥ 10% or ΔDys2+ ≥ 10%, the patient chooses proton. If the ΔNTCP of grade 2 complications alone does not exceed 10%, the decision is made by combining two grade 2 complications. If grade 2 complications cannot be made, the ΔNTCP of grade 3 complications of XT and PT need to be compared, and the threshold is 5%.

### 2.6. Performance Evaluation

In order to evaluate the accuracy of dose prediction, we compared the prediction results with the results of MP and the evaluation indicators were consistent with the results of manual plan according to clinical requirements.

We selected key parameters of the organs at risk related to the calculation of NTCP values as the prediction targets of deep learning, including the mean dose of affected parotid and contralateral parotid, the mean dose of the lower submandibular glands, the mean dose of the oral cavity, the mean dose of the upper pharyngeal constrictor muscle, the mean dose of the middle pharyngeal constrictor muscle, and the mean dose of the inferior pharyngeal constrictor muscle. We compared the key parameters of the predictive model output with the results of the MP. Mean absolute error (MAE) and mean error were employed to evaluate the accuracy of dose predictions by the DL model shown in Equations (6) and (7).

(6) $$MAE=\frac{1}{N}\times \frac{\sum_{j=1}^{N}\frac{1}{M}\sum_{i=1}^{M}\left|D_{\mathrm{Pr}ed}(i)-D_{MP}(i)\right|}{\mathrm{Pr}escription\;dose}\times 100\%$$

(7) $$MAE=\frac{1}{N}\times \frac{\sum_{j=1}^{N}\frac{1}{M}\sum_{i=1}^{M}\left(D_{\mathrm{Pr}ed}(i)-D_{MP}(i)\right)}{\mathrm{Pr}escription\;dose}\times 100\%$$

where i is the index of the voxel in each ROI for each patient and M is the total number of involved voxels. D_Pred_(i) and D_MP_(i) are the predicted and manual plan (MP) dose of a voxel i, respectively, j is the index of the case, and N is the total number of cases in the test set.

The prediction results of deep learning and the results of MP were input into the NTCP model to calculate the NTCP values of xerostomia and dysphagia, and the difference analysis was performed to evaluate the accuracy of the deep learning model in predicting complications

The performance of the decision tool was quantified in terms of the mean accuracy [accuracy = (TP + TN)/(TP + FP + FN + TN)], sensitivity [sensitivity = TP/(TP + FN)], specificity [specificity = TN/(TN + FP)], F-score [F-score = 2TP/(2TP + FN + FP)], and area under the receiver operator characteristic curve (ROC) (AUC), where TP is true positive, TN is true negative, FP is false positive, and FN is false negative. A positive result means the selection of PT, while a negative result means the adoption of XT.

### 2.7. Statistical Analysis

The normality of the samples and the equality of variances were tested. The parametric paired *t*-test with a two-tailed approach is adopted to evaluate whether the difference between the manual plan and DL is statistically significant. The criterion of *p* < 0.05 was adopted.

## 3. Results

### 3.1. Dose Prediction Accuracy

**Figure 4:**
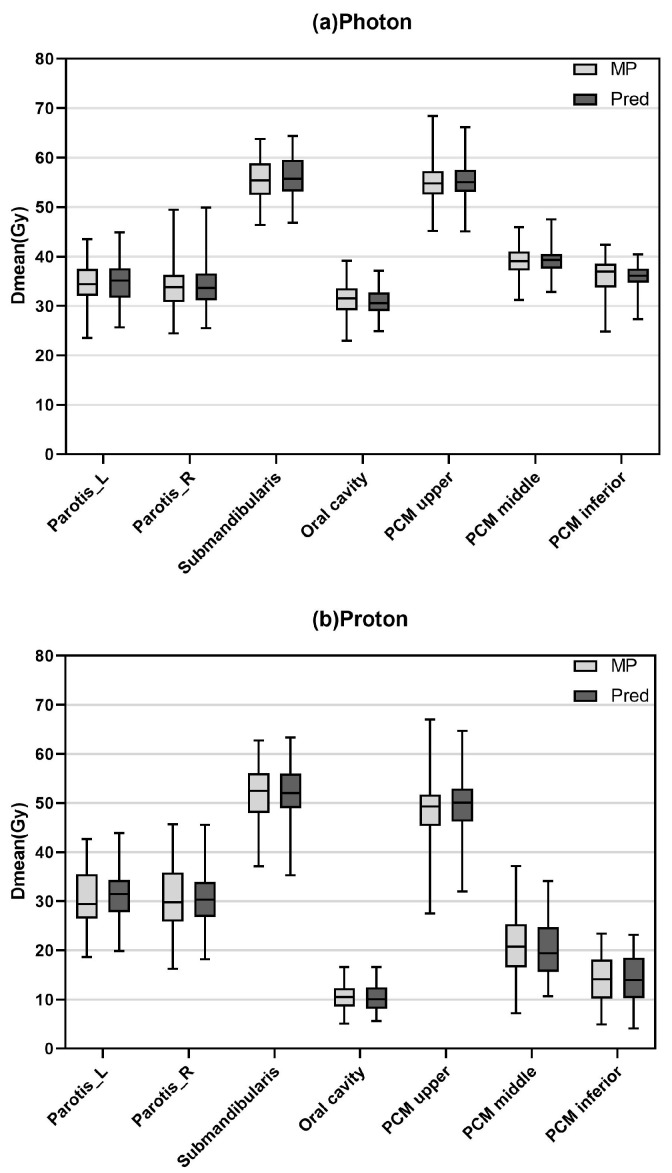
Boxplots displaying the mean dose of organ at risk across the 48 patients; on the left, the mean dose of MP and Pred for photon, and on the right, the mean dose of MP and Pred for photon. The dark line in the middle of the box indicates the median of the box indicates and the lower and upper edges of the box indicate the 25th and 75th percentile, respectively. The whiskers indicate the range of the data. Parotid_L—parotid left; Parotid_R—parotid right; MP—manual plan; Pred—prediction.

We accurately predicted the mean dose (Dmean) of key organs such as parotid, submandibular, oral cavity, and pharyngeal ducts in photon and proton therapy regimens and compared it with the values of the MP. The MAE for each organ at risk in the photon and proton plans showed the good performance of the dose prediction model, as shown in Table 2. The results demonstrate robust model performance across most organs shown in Figure 4. The predicted doses for both photon and proton groups were comparable to those for MP. The ME showed that the predicted dose is not biased towards one direction compared to the manual plan dose.

### 3.2. NTCP Modeling Accuracy

Table 3 shows the results of mean NTCP for photon and proton. For xerostomia grade 2, the absolute deviation between predicted and MP NTCP values was minimal, at 0.85% for photon therapy (*p* = 0.003) and 0.75% for proton therapy (*p* = 0.082), with non-significant statistical differences. In predicting xerostomia grade 3, the absolute deviations remained low, at 1.11% (*p* = 0.003) and 0.90% (*p* = 0.109), respectively, demonstrating the model’s reliability in predicting xerostomia outcomes. For dysphagia grade 2, the prediction of photon and proton NTCP is stable and good with the absolute deviation −1.00% (*p* = 0.303) and −0.85% (*p* = 0.426). For dysphagia grade 3, the absolute deviation for the prediction of photon and proton NTCP is larger, −1.81% (*p* = 0.305) and −4.76% (*p* = 0.056), but with no significant difference.

### 3.3. Decision-Making Accuracy

**Figure 5:**
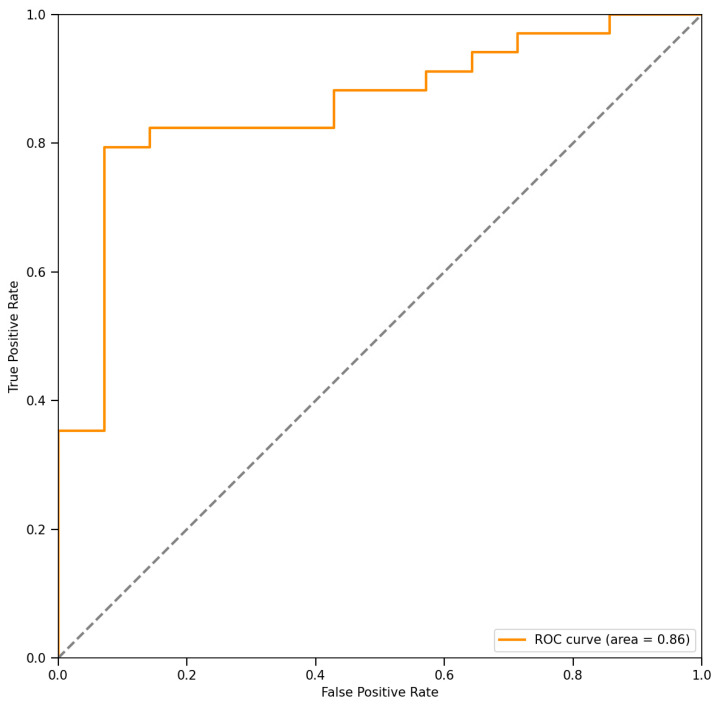
The receiver operating characteristic curve (ROC) and area under curve (AUC) values of the decision tool.

By comparing the predicted outcomes of our tool against the true values derived from MP decisions, we evaluated the predictive performance of our system. In this study, 33 of the 48 patients were referred to proton therapy, as shown in Figure 5. Results indicate that among all evaluated cases, discrepancies with MP decisions were observed in only three cases (6.3% of the total), with one erroneous recommendation for proton therapy and two for photon therapy. Despite these few exceptions, our decision tool demonstrated a high level of predictive accuracy, achieving an overall accuracy rate of 93.7%. Further analysis revealed a sensitivity of 97.1%, signifying the effective identification and recommendation of the majority of patients who should undergo proton therapy. The specificity of 92.3% reflected the tool’s accuracy in excluding patients unsuitable for proton therapy. Additionally, the high F1 score of 95.7% validated the system’s exceptional performance in balancing precision and recall. The ROC presented in Figure 5, along with its AUC value of 0.86, underscores the good diagnostic performance of our decision tool in distinguishing indications for proton versus photon therapy, showcasing the potential and value of AI in radiotherapy treatment decision-making.

## 4. Discussion

In this study, we proposed a clinical decision-making tool for nasopharyngeal cancer, which used deep learning to predict the dose of the patient’s photon plan and proton plan and input the average dose prediction of the relevant organs at risk into the Dutch NIPP model for NTCP prediction, referring to the decision process given by NIPP to make decisions about the treatment technology of the patient. By comparing the predictions with the ground truth from MP, our study demonstrated the high accuracy of the tool in both dose prediction and NTCP assessment, underscoring its potential in clinical decision support.

In the decision-making process provided by NIPP, the xer2+ NTCP value of photon is first referred to. If it is greater than 10%, then the ΔNTCP value of xer2+ is obtained by subtraction with the proton. Patients who did not meet the criteria continued to be compared for Dys2+. The decision process in this study (see Figure 3) does not reflect this process because the NTCP value of xer2+ in 48 photon projects is greater than 10%, and the decision process automatically moves to a single process. This result is related to the target area of patients. For patients with bilateral lymphoma nasopharyngeal carcinoma selected in this study, the target area almost includes bilateral parotid glands. In order to cover the dose of the target area, the average dose of parotid glands will inevitably be high due to the photon plan of two full arcs irradiation. In the proton program, although we tried three fields (0°, 135°, and 315°) to avoid irradiation of the parotid gland, the parotid gland could not significantly reduce the dose due to too much overlap with the target area, which resulted in no significant reduction in the proton program Xer2+ used in this study, so that all 50 patients were moved to the next decision process. This suggests that the position relationship between the target area and the organ at risk affects the decision-making process, but it does not indicate the failure of the decision-making process. According to the subsequent decision-making process, we correctly chose proton therapy for patients who may benefit from clinical treatment.

Notably, despite the tool’s impressive performance, there were three cases (6.3%) where the recommended treatment modalities did not align with the MP decisions. Further examination of these cases revealed that the ΔNTCP prediction for dysphagia grade 2 (9.94%, 9.56% and 10.88%) was situated near the decision threshold of 10% in all mismatched instances. This observation highlights the sensitivity of the model to minute errors when NTCP predictions hover around critical values, potentially leading to opposing treatment recommendations. It also shows that the threshold selection of ΔNTCP can affect the decision result. The decision tool developed by Camille et al. [19] is highly dependent on thresholds, so they explored values in the range of 1–20% and reached accuracies in the range of 92.1–100%. The clinician has access to the dose distribution predicted by the DL model and has control over the choice of the NTCP model and the threshold at which a patient should be redirected to PT. This work is worth learning from to improve the decision-making process.

In the proton plan, the greatest dose reduction was seen in the organs associated with dysphagia like oral cavity, PCM middle, and PCM inferior. The result in dysphagia showed a high gain in NTCP. This is easily understandable as there are numerous OARs, such as the three pharyngeal constructor muscles or oral cavity, that are located in the central area and cannot be spared with XT plans [24]. For the 33 patients selected for proton therapy in this study, 31 were selected based on Dys2+ ≥ 10%. For the studied version of the NIPP (v2.2), manipulation of the Dys2+ model had the largest impact on treatment indications. This is consistent with the results of Artuur [20]. This suggests that we can optimize the process for clinical decision-making in nasopharyngeal cancer.

Model-based methods typically require generating at least one plan for each compared mode (XT and PT) and then providing the corresponding dose distribution to the NTCP model. Generating plans that meet clinical standards requires manual operation, takes a long time to generate, and requires high skills from dosimeters, especially proton plans [24]. Proton therapy is still sufficiently uncommon to be largely absent from most physicians’ clinical training [13]. But the field is growing fast enough [20] that there is a steady need for new physicians requiring knowledge transfer from those with prior experience [25]. The decision tool we developed can automatically segment the patient’s CT image to generate their anatomical structure, which can be used as an input to output the photon and proton dose distribution in a very short time, and give the decision result based on the NTCP model for clinicians’ reference. This significantly reduces patient wait times, and the accuracy of decision-making tools can also improve doctors’ diagnoses. At the same time, these advanced tools can be transferred to lower-level radiotherapy institutions to improve their diagnosis and treatment level.

The method proposed in this study has some limitations. First of all, we used a single case of bilateral lymphoma of nasopharyngeal carcinoma in our center. For other head and neck cases, the reliability of the model needs to be verified and corresponding modifications made. Secondly, the decision-making tool we proposed is based on the protocol of the National Proton Center of the Netherlands, and whether the proposed scheme or the setting of the threshold is in line with the actual situation of our country remains to be further explored. Third, our TPS can only set range uncertainty and does not incorporate other robustness optimization functions. To ensure consistency in comparison with photon plan, our proton plan was optimized based on PTV. In the future, we will employ CTV-based robustness optimization to further improve the proposed proton–photon decision-making method.

## 5. Conclusions

We developed a fully automated decision tool to select patients for proton therapy that predicts XT and PT dose distributions using only patient CT image data, predicts xerostomia and dysphagia probability using predicted critical organ mean doses, and makes decisions based on NIPP to select patients likely to benefit from proton therapy, with 93.8% accuracy. The tool has a short decision-making time and high accuracy, reduces the waiting time of patients, and improves the diagnostic efficiency of doctors.

## Figures and Tables

**Table 1:** Overview of the clinical dose criteria.

Structure	Dosimetric Parameter	Per Protocol
Target		
PGTV74	V73.92 Gy [%]	≥95
PGTV70	V69.96 Gy [%]	≥95
PTV60	V60.06 Gy [%]	≥95
OAR		
Spinal Cord PRV	Dmax [Gy]	≤40
Brain stem PRV	Dmax [Gy]	≤40
Lens L	Dmax [Gy]	≤9
Lens R	Dmax [Gy]	≤9
Optic Nerve L	Dmax [Gy]	≤66
Optic Nerve R	Dmax [Gy]	≤66
Optic Chiasm	Dmax [Gy]	≤66
Parotid L	V30 Gy [%]	≤50
Parotid R	V30 Gy [%]	≤50
Larynx	Dmax [Gy]	≤40
Trachea	Dmax [Gy]	≤40

**Table 2:** The MAE and ME of OARs for photon and proton.

	Parotid_L (%)	Parotid_R (%)	Submandibularis (%)	Oral Cavity (%)	PCM U (%)	PCM M (%)	PCM I (%)
MAE							
Photon	4.49 ± 1.54	4.63 ± 1.48	3.00 ± 0.82	3.91 ± 1.62	3.19 ± 0.81	2.77 ± 1.21	3.50 ± 1.68
Proton	4.22 ± 1.23	4.15 ± 1.67	3.95 ± 1.50	2.78 ± 1.00	4.28 ± 1.09	4.62 ± 2.59	3.88 ± 1.92
ME							
Photon	0.0060 ± 0.0044	0.0068 ± 0.0042	0.0094 ± 0.0022	−0.0024 ± 0.0045	0.0008 ± 0.0025	0.0047 ± 0.0035	−0.0003 ± 0.0049
Proton	0.0056 ± 0.0052	0.0051 ± 0.0052	0.0007 ± 0.0044	−0.0071 ± 0.0029	0.0062 ± 0.0040	−0.0009 ± 0.0069	−0.0065 ± 0.0049

**Table 3:** The mean NTCP for photon and proton.

		Mean NTCP (%)		*p*
		MP	Prediction	
Xer2+	XT	46.98 ± 2.44	47.38 ± 2.32	0.003
	PT	43.72 ± 3.53	44.05 ± 3.10	0.082
Xer3+	XT	13.46 ± 0.97	13.61 ± 0.93	0.003
	PT	12.22 ± 1.32	12.33 ± 1.16	0.109
Dys2+	XT	17.00 ± 2.76	16.83 ± 2.33	0.303
	PT	5.87 ± 1.59	5.82 ± 1.42	0.426
Dys3+	XT	3.87 ± 0.96	3.80 ± 0.75	0.305
	PT	0.63 ± 0.30	0.60 ± 0.25	0.056

## Data Availability

Research data are not available at this time.

## Abbreviations

**XT**	photon therapy
**PT**	proton therapy
**NIPP**	Netherlands’s National Indication Protocol Proton therapy
**NCC**	convolutional neural network
**DL**	deep learning
**NTCP**	normal tissue complication probability
**RT**	radiation therapy
**IMRT**	intensity-modulated radiation therapy
**VMAT**	volumetric modulated arc therapy
**IMPT**	intensity-modulated proton therapy
**OAR**	organs at risk
**MRI**	magnetic resonance imaging
**NPC**	nasopharyngeal carcinoma
**TPS**	treatment planning system
**PMC U**	pharyngeal constrictor muscles upper
**PMC M**	pharyngeal constrictor muscles middle
**PMC I**	pharyngeal constrictor muscles inferior
**DPTV**	contours and distance map
**COM**	combined model
**PL**	parotid left
**PR**	parotid right
**OC**	oral cavity
**MP**	manual plan
**MAE**	mean absolute error
**Dmean**	mean dose
**AUC**	area under curve
**ROC**	operating characteristic curve
**Xer2+**	xerostomia ≥ grade 2
**Xer3+**	xerostomia ≥ grade 3
**Dys2+**	dysphagia ≥ grade 2
**Dys3+**	dysphagia ≥ grade 3
